# Curcumin Attenuates Damage to Rooster Spermatozoa Exposed to Selected Uropathogens

**DOI:** 10.3390/pharmaceutics15010065

**Published:** 2022-12-26

**Authors:** Eva Tvrdá, Michaela Petrovičová, Filip Benko, Michal Ďuračka, Lucia Galovičová, Tomáš Slanina, Miroslava Kačániová

**Affiliations:** 1 Institute of Biotechnology, Faculty of Biotechnology and Food Sciences, Slovak University of Agriculture in Nitra, Tr. A. Hlinku 2, 949 76 Nitra, Slovakia; 2 Institute of Applied Biology, Faculty of Biotechnology and Food Sciences, Slovak University of Agriculture in Nitra, Tr. A. Hlinku 2, 949 76 Nitra, Slovakia; 3 Department of Neuroscience, Second Faculty of Medicine (2. LF UK), V Úvalu 84, 150 06 Prague, Czech Republic; 4 AgroBioTech Research Centre, Slovak University of Agriculture in Nitra, Tr. A. Hlinku 2, 949 76 Nitra, Slovakia; 5 Department of Fruit Science, Viticulture and Enology, Faculty of Horticulture and Landscape Engineering, Slovak University of Agriculture in Nitra, Tr. A. Hlinku 2, 949 76 Nitra, Slovakia; 6 Department of Bioenergetics, Food Analysis and Microbiology, Institute of Food Technology and Nutrition, University of Rzeszow, Cwiklinskiej 1, 35-601 Rzeszow, Poland

**Keywords:** curcumin, bacterial contamination, roosters, semen storage, *Salmonella*, *Escherichia*, *Pseudomonas*, antibiotics

## Abstract

Artificial insemination, as an essential pillar of the modern poultry industry, primarily depends on the quality of semen collected from stud roosters. Since the collection and storage of ejaculates is not a sterile process, antimicrobial agents have become essential supplements to semen extenders. While the use of traditional antibiotics has been challenged because of rising bacterial resistance, natural biomolecules represent an appealing alternative because of their antibacterial and antioxidant properties. As such, this study strived to compare the effects of 50 μmol/L curcumin (CUR) with 31.2 µg/mL kanamycin (KAN) as a conventional antibiotic on rooster sperm quality in the presence of *Salmonella enterica*, *Escherichia coli* and *Pseudomonas aeruginosa*. Changes in sperm structural integrity and functional activity were monitored at 2 and 24 h of culture. Computer-assisted semen analysis revealed significant sperm motility preservation following treatment with KAN, particularly in the case of *Salmonella enterica* and *Pseudomonas aeruginosa* (*p* < 0.001) after 24 h. On the other hand, CUR was more effective in opposing ROS overproduction by all bacteria (*p* < 0.05), as determined by luminol-based luminometry, and maintained sperm mitochondrial activity (*p* < 0.001 in the case of *Salmonella enterica*; *p* < 0.05 with respect to *Escherichia coli* and *Pseudomonas aeruginosa*), as assessed by the fluorometric JC-1 assay. The TUNEL assay revealed that CUR readily preserved the DNA integrity of rooster sperm exposed to *Salmonella enterica* (*p* < 0.01) and *Escherichia coli* (*p* < 0.001). The bacteriological analysis showed higher efficiency of KAN in preventing the growth of all selected bacterial species (*p* < 0.0001) as opposed to CUR. In conclusion, CUR provided protection to rooster spermatozoa against alterations caused by uropathogens, most likely through its antioxidant activity. Hence, CUR supplementation to poultry semen extenders in combination with properly selected antibacterial substances may become an interesting strategy in the management of bacterial contamination during semen storage.

## 1. Introduction

Since the end of the 19th century when Ilya Ivanovich Ivanov started experimenting with semen collected from the ductus deferens of roosters, artificial insemination (AI) has become an indispensable tool in the poultry industry [1]. This biotechnological approach enables more efficient utilization of genetically superior males with high reproductive performance, leading to desirable fertilization rates and hatchability, which are unachievable under natural mating conditions [1,2]. This will in turn decrease production costs by reducing the number of roosters, thereby saving space, feed, maintenance, and operating expenses [1]. Nevertheless, it must be acknowledged that the success of AI is fundamentally dependent on semen quality, which is needed to maximize reproductive output while minimizing the wastage of an animal’s investment in producing male gametes [3]. In the meantime, extended ejaculates may be affected by a vast array of endogenous and exogenous factors, including bacterial contamination, which may compromise the shelf life of insemination doses, fertility rates and hatchability [4,5].

The proximity of the gastrointestinal and reproductive systems of birds predisposes avian semen to bacterial contamination. It has been previously reported that virtually all poultry ejaculates contain bacteria, among which *Salmonella* spp., *Pseudomonas* spp., *Campylobacter* spp., *Escherichia coli* and *Clostridium* spp. are the most common [5,6,7,8,9]. If necessary precautions are not taken during semen processing, most extended semen specimens will present with bacterial contamination [1,2,10], with negative consequences on sperm survival and fertilization ability. Sperm agglutination and immobilization, morphological alterations, DNA breakage and oxidative damage to male gametes have often been linked to the occurrence of bacteria in ejaculates of several mammalian and avian species [5,9,11,12,13]. Moreover, uropathogenic bacteria present in insemination doses may be easily transmitted to females and thus negatively impact production, either by causing infections to the flock or by contributing to a decreased laying frequency and hatchability [4,14,15]. Finally, harmful bacteria may be transmitted to poultry products with potentially negative effects on human health if consumed [15,16].

Since rooster ejaculates are highly concentrated and viscous, semen extenders have become routine in chicken breeding [1,10]. Avian spermatozoa are inherently vulnerable to the cold shock associated with sperm cryopreservation, which is why temperatures ranging between 4 and 10 °C are considered to be suitable for the storage of poultry semen [1,17]. Nevertheless, in order to ensure an optimal environment for ex vivo sperm survival, the extenders are usually rich in nutrients, which on the other hand may favor bacterial growth if a contaminated semen sample is processed and stored [6,9,10,11,18].

While antibiotics are generally the first choice to prevent and/or counteract bacterial growth in extended semen [6,19], andrologists have shifted their attention to alternative biomolecules that could improve sperm viability by avoiding any potentially negative effects of antibiotics on male gametes, which may range from motility inhibition and higher incidence of pathological spermatozoa to direct spermatotoxicity [20,21]. Furthermore, substances derived from natural resources could be beneficial for production safety through deceleration of the spread of antibiotic resistance [20,21,22]. According to previous reports, curcumin (1,7-bis (4-hydroxy-3-methoxyphenyl)-1,6-heptadiene-3,5-dione)) (CUR), a vibrant yellow curcuminoid found primarily in Turmeric (*Curcuma longa*), has exhibited significant beneficial effects on the motility, membrane stability and mitochondrial activity of spermatozoa subjected to liquid storage or cryopreservation in bulls [23], rabbits [24], boars [25], goats [26] and roosters [27]. CUR is an effective scavenger of superoxide, nitrogen dioxide and hydroxyl radicals, as well as a strong inhibitor of lipid peroxidation [23,26,28]. At the same time, previous studies have emphasized the versatile antibacterial activity of CUR against G^+^ as well as G^-^ bacteria, such as *Staphylococcus aureus (S. aureus)*, *Staphylococcus haemolyticus (S. haemolyticus)*, *Enterococcus faecalis (E. faecalis)*, *Escherichia coli (E. coli)*, *Proteus mirabilis (P. mirabilits)* and *Pseudomonas aeruginosa (P. aeruginosa)*, most likely through its ability to inhibit bacterial virulence factors, adhesion to host cells and biofilm formation [29,30,31].

Although previous research suggests that CUR exhibits beneficial effects on sperm quality under in vitro conditions while at the same time having antibacterial properties, its behavior under in vitro induced bacterial contamination of avian semen has not yet been investigated. As such, this study was designed to evaluate the in vitro efficiency of CUR to prevent or counteract damage to rooster spermatozoa exposed to *Salmonella enterica*, *Escherichia coli* and *Pseudomonas aeruginosa*, which are known to cause uropathogenic infections and subsequent loss of semen quality in poultry breeding. At the same time, we compared the observed effects of CUR with kanamycin (KAN) as a representative of antibiotics conventionally used in animal biotechnologies [31,32] in order to estimate the mechanism by which both substances prevent damage to male gametes caused by bacterial action. 

## 2. Materials and Methods

### 2.1. Semen Samples

Sixty semen samples for this study were obtained by cloacal massage of 20 sexually mature Lohmann Brown roosters housed by a local broiler breeding company (Liaharenský podnik Nitra, a.s., Párovské Háje, Slovakia). Pure semen was collected in sterile collection syringes and subsequently transported to the laboratory in a Mini Bio Isotherm system (M&G Int, Renate, Italy). Once in the laboratory, the ejaculates were assessed for baseline sperm count and motility, and heterospermic specimens were prepared in order to obtain a suitable sperm concentration and motility of at least 65% for subsequent in vitro experiments. Overall, 20 heterospermic samples were used for the experiments.

### 2.2. Bacteria

*Salmonella enterica* (*S. enterica*), *E. coli* and *P. aeruginosa* were isolated from pure rooster semen prior to the in vitro experiments. Briefly, 100 μL quantities of each sample were inoculated onto selected sterile agars (blood agar base no. 2; Gassner agar, NutriSelect^®^ basic; soybean casein digest agar; Merck, Darmstadt, Germany) and incubated under aerobic conditions at 36 ± 2 °C for 24 h. Following isolation and purification of the bacterial colonies, these were identified using the Biotyper MALDI-TOF (matrix-assisted laser desorption–ionization time-of-flight) mass spectrometer (MS; Brucker Daltonics, Bremen, Germany) implementing the Microflex LT instrument and flexControl software version 3.4. The spectra obtained by the mass spectrometer were processed with the MALDI Biotyper Bruker Taxonomy database (Bruker Daltonics, Bremen, Germany), according to Lenický et al. [5] and Duracka et al. [31]. Bacterial isolates previously stored at −80 °C were hydrated in their respective growth media (SEB broth, Sigma-Aldrich, St. Louis, MO, USA, in the case of *S. enterica*; EC Broth, Sigma-Aldrich, St. Louis, MO, USA, for *E. coli*; *Pseudomonas* broth, Sigma-Aldrich, St. Louis, MO, USA, for *P. aeruginosa*) and cultured for 24–48 h at 36 °C 1 week before the start of the experiment. In order to ensure optimum growth conditions, 1 mL of each bacterial culture was transferred into 9 mL of its respective broth every 24 h. On the day of the experiments, small amounts of the broths containing the various bacterial cultures were added to Glutac semen extender for poultry sperm (AMP-Lab GmbH, Münster, Germany) and the resulting bacterial loads were adjusted to 10^8^ CFU/mL with pure extender while simultaneously monitoring the bacterial concentrations with the help of a DEN-1 McFarland densitometer (Grant-Bio, Cambridge, UK).

### 2.3. Media Preparation

The final concentration of 31.2 µg/mL kanamycin (KAN; Sigma-Aldrich, St. Louis, MO, USA) as the conventional antibiotic for our comparative analysis was selected on the basis of collected evidence on its biological activity and potential toxicity towards bacterial or eukaryotic cells [31,32], as well as previous validation experiments in our laboratory. In the case of curcumin (CUR), 50 μmol/L (Sigma-Aldrich, St. Louis, MO, USA) was selected as an optimal dose for ensuring a desirable level of rooster sperm survival under in vitro conditions during previous standardization experiments. The stock solution of 3.12 mg/mL KAN was prepared using the Glutac extender, while the stock solution of 10 mmol/L CUR was prepared with dimethyl sulfoxide (DMSO, Sigma-Aldrich, St. Louis, MO, USA). Respective aliquots of KAN or CUR stock solutions were added to the Glutac extender to reach the final concentrations of both substances shortly before bacteria and spermatozoa were added to the cultures. Additionally, DMSO was added to the extender for the controls and experimental groups carrying KAN to reach its final concentration of 0.5% across all groups established in the study.

### 2.4. Sperm Cell Preparations

In order to purify viable spermatozoa from bacteria and somatic cells, as well as spermatocytes, immature spermatids and non-viable sperm cells, we used Percoll Plus^®^ (Merck, Darmstadt, Germany) gradient centrifugation. First, 400 μL of each sample diluted with 1 mL PBS (phosphate-buffered saline; Sigma-Aldrich, St. Louis, MO, USA) was centrifuged at 805× *g* for 5 min. The supernatant was removed, and the cells were resuspended in 1 mL PBS. A quantity of 1 mL of the mixture was carefully placed on the top of the gradient medium consisting of 1.5 mL 90% Percoll and 1.5 mL 45% Percoll, both pre-warmed to 37 °C. Following centrifugation (805× *g*, 30 min), the lower layer was transferred into a 2 mL tube and centrifuged again at 805× *g* for 5 min. Viable spermatozoa deposited on the surface were separated from the medium, washed twice with PBS and subjected to a final centrifugation at 805× *g* for 5 min [33]. Each sample was then diluted in the pre-established Glutac media containing bacteria and biomolecules at a ratio of 1:100 and stored for 24 h at 4 °C. Specific analyses were carried out immediately following dilution (negative control exclusively), after 2 h and 24 h of storage.

In this study, the negative control contained spermatozoa exclusively. For the positive controls, respective controls were established for each bacterium without the presence of any bioactive molecule. 

### 2.5. Sperm Motility

The percentage of motile spermatozoa was assessed with the CASA (computer-assisted sperm analysis) system (version 14.0 TOX IVOS II, Hamilton-Thorne Biosciences, Beverly, CA, USA). Each diluted sample (7 µL) was placed in a Makler counting chamber (10 µm depth; Sefi Medical Instruments, Haifa, Israel), which was then inserted into a pre-heated plate set at 37 °C. The computer system evaluated sperm motion by automatically scanning 10 different microscopic fields within the chamber. Sperm motility is expressed as the percentage (%) of cells moving faster than 5 μm/s [5].

### 2.6. Membrane Integrity

The integrity of sperm plasma membranes was determined with the eosin–nigrosin colorimetric approach. A quantity of 5 μL of each semen specimen was mixed with 10 μL eosin (Eosin Y; Sigma-Aldrich, St. Louis, MO, USA) and subsequently 10 μL of the nigrosin contrast dye (Sigma-Aldrich, St. Louis, MO, USA) on a microscopic slide. A second slide was used to prepare a smear which was then allowed to dry at room temperature. The slides were evaluated using the Leica DM IL LED inverted microscope (Leica Microsystems, Wetzlar, Germany). Three hundred cells were counted on each slide by one experienced observer, and the proportion of cells with intact membranes is expressed as a percentage (%) [34].

### 2.7. Acrosome Integrity

The integrity of the acrosome was determined using a double-staining method based on fast green and rose bengal. Each sample (20 μL) was mixed with 20 μL of a staining solution comprising both stains (Sigma-Aldrich, St. Louis, MO, USA) and incubated at room temperature for 70 s. Subsequently, 10 μL of the mixture was smeared on a slide and air-dried. All slides were evaluated by one experienced observer under a Leica DM IL LED microscope by counting 300 cells. Acrosome integrity was expressed as the proportion of spermatozoa with an intact acrosomal cap (%) [5].

### 2.8. Mitochondrial Membrane Potential

Mitochondrial activity was evaluated with the JC-1 Mitochondrial Membrane Potential Assay kit (Cayman Chemical, Ann Arbor, MI, USA), taking advantage of the lipophilic, light-sensitive cationic dye JC-1 (5.5′,6.6′-tetrachloro-1,1′,3,3′-tetraethylbenzimidazolylcarbocyanine iodide), which was mixed (5 μL) with 100 μL of the sample. Following incubation (37 °C, 30 min), the samples were centrifuged (150× *g*, 25 °C, 5 min) and washed twice with a washing buffer provided with the kit. Finally, the samples were transferred onto dark 96-chamber plates that were processed with the combined GloMax-Multi+ spectro-fluoro-luminometer (Promega, Madison, WI, USA). The resulting mitochondrial membrane potential is expressed as the ratio of JC-1 complexes to JC-1 monomers (green/red ratio) [31].

### 2.9. DNA Integrity

The APO-DIRECT^TM^ TUNEL assay kit (BD Biosciences; Franklin Lakes, NJ, USA) was used to assess sperm DNA fragmentation. One million cells collected from each specimen were fixed in 4% paraformaldehyde (Centralchem, Bratislava, Slovakia). Following sample incubation on ice for 1 h, the cells were washed 3 times in PBS and stored in 70% ice-cold ethanol (Centralchem, Bratislava, Slovakia) at −20 °C overnight. The next day, the cells were washed, labeled with the DNA labeling solution containing terminal deoxynucleotidyl transferase (TdT), fluorescein–deoxyuridine triphosphate rinse, TdT reaction buffer and distilled water (BD Biosciences; Franklin Lakes, NJ, USA), and centrifuged (805× *g*, 5 min) twice. Each sample was then counterstained with DAPI (4′,6-diamidino-2-phenylindole; Sigma-Aldrich, St. Louis, MO, USA; 1 μmol/L in PBS), transferred to a dark 96-well plate and read using the Glomax Multi+ spectro-fluoro-luminometer. The proportion of cells with fragmented DNA is expressed as a percentage (%) [5].

### 2.10. ROS Production

To determine the extent of oxidative stress, the quantities of ROS produced by the control and experimental samples were quantified by the chemiluminescent assay based on the ability of luminol (5-amino-2,3-dihydro-1,4-phthalazinedione; Sigma-Aldrich, St. Louis, MO, USA) to interact with reactive intermediates. The samples (100 μL) were pipetted onto clear 96-well plates, carrying blank (100 μL PBS), negative control (100 μL PBS) and positive control (100 μL PBS, 12.5 μL 30% hydrogen peroxide (H_2_O_2_; Sigma-Aldrich, St. Louis, MO, USA)). Subsequently, the samples and controls were treated with 2.5 μL of luminol working solution and the resulting light signal was monitored in 15 consecutive one-minute-long cycles using the Glomax Multi+ spectro-fluoro-luminometer. The results are expressed as relative light units (RLU)/s/10^6^ spermatozoa [31].

### 2.11. Bacteriological Analysis

All controls and experimental groups were subjected to a follow-up microbiological analysis to verify the effectivity of the Percoll gradient to eliminate bacteria from semen (absence of any bacteria in the groups containing spermatozoa exclusively) and to confirm the exclusive presence of the bacteria selected for the experiments in their respective control or experimental groups. The analysis consisted of bacterial cultures on selective agars and MALDI-TOF MS identification, as described previously.

Positive controls and experimental samples that tested positive for the presence of *Salmonella enterica*, *Escherichia coli* and *Pseudomonas aeruginosa* exclusively were further cultured using selective agars mentioned earlier at 37 °C for 48–72 h, and the plate dilution method was used to quantify the bacterial loads (log_10_ CFU/mL) of the respective bacteria in order to verify the ability of KAN or CUR to inhibit bacterial growth in the sperm–bacteria co-culture.

### 2.12. Statistical Analysis

Statistical analysis was carried out with the GraphPad Prism program (version 8.4.4 for Mac; GraphPad Software Incorporated, La Jolla, CA, USA). The results are expressed as means ± standard deviations. Differences between the groups were analyzed using one-way ANOVA followed by the Tukey multiple comparisons test, designed to compare the means of three or more independent samples simultaneously. The comparative analysis was performed in the following sequence:

- The negative control was compared to the positive controls containing bacteria without the presence of CUR or KAN.
- Experimental groups were compared to their respective positive controls containing bacteria.

Statistical significance was set at *p* < 0.05 (*); *p* < 0.01 (**); *p* < 0.001 (***); and *p* < 0.0001 (****).

## 3. Results

### 3.1. Sperm Motility

**Figure 1:**
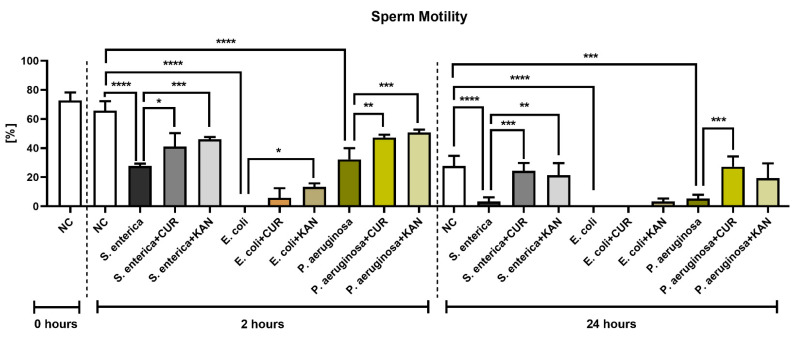
The effect of curcumin and kanamycin on rooster sperm motility (%) during induced bacterial contamination. *p* < 0.05 (*), *p* < 0.01 (**), *p* < 0.001 (***), *p* < 0.0001 (****). NC—negative control, CUR—curcumin, KAN—kanamycin.

The CASA analysis following 2 h of incubation revealed an abrupt decrease in sperm motility in all positive controls containing the selected bacterial strains; the decreases were significantly different in comparison with the negative control (*p* < 0.0001; Figure 1). Interestingly, while in the case of *Salmonella enterica* (*S. enterica*) and *P. aeruginosa*, a minor portion of spermatozoa remained motile, complete motility inhibition was observed in the control group containing *E. coli*. Both CUR as well as KAN were able to stabilize the sperm motion decline in the experimental groups in comparison with their respective controls. In all established experimental groups, the motility was higher when KAN was used as a supplement.

Further motility deterioration, particularly in the positive controls, was observed after 24 h. In fact, the motility rate in the controls containing *S. enterica* and *P. aeruginosa* was almost null (*p* < 0.0001 with respect to *S. enterica*; *p* < 0.001 in the case of *P. aeruginosa*; in comparison with the negative control), while only non-motile spermatozoa were present in the *E. coli* control (*p* < 0.0001 in comparison with the negative control).

While a minor preservation of sperm motion was recorded in the experimental group comprising *E. coli* and KAN, no motility improvement was observed in the case of CUR. Inversely, a higher motility proportion was observed in the experimental groups carrying *S. enterica* and *P. aeruginosa* that had been supplemented with CUR in comparison to their respective positive controls (*p* < 0.001). A significantly higher sperm motility was also observed in the case of *S. enterica* and KAN (*p* < 0.01).

### 3.2. Membrane Integrity

**Figure 2:**
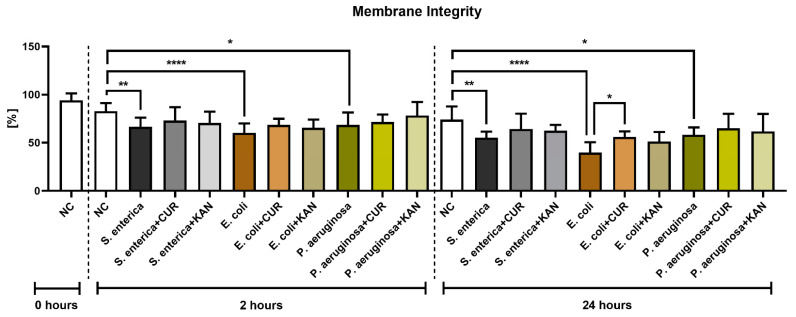
The effect of curcumin and kanamycin on rooster sperm membrane integrity (%) during induced bacterial contamination. *p* < 0.05 (*), *p* < 0.01 (**), *p* < 0.0001 (****). NC—negative control, CUR—curcumin, KAN—kanamycin.

A significant decrease in membrane integrity was observed after 2 and 24 h in all positive controls when compared with the negative control (*p* < 0.01 in the case of *S. enterica*; *p* < 0.0001 for *E. coli*; *p* < 0.05 for *P. aeruginosa*; Figure 2). While a slight improvement in membrane integrity was observed following CUR or KAN administration at 2 h, no significant differences were observed between the positive controls and experimental groups. After 24 h, a significant stabilization of sperm plasma membranes was observed only in the case of spermatozoa exposed to CUR in the presence of *E. coli* (*p* < 0.05).

### 3.3. Acrosome Integrity

**Figure 3:**
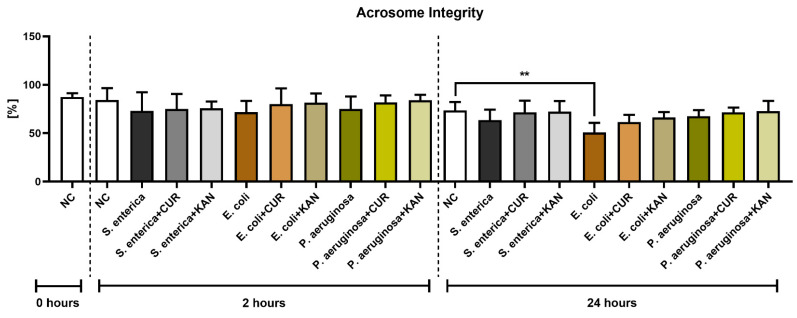
The effect of curcumin and kanamycin on rooster sperm acrosome integrity (%) during induced bacterial contamination. *p* < 0.01 (**). NC—negative control, CUR—curcumin, KAN—kanamycin.

Similar to membrane integrity, a decline in the stability of acrosomal structures was observed in all positive controls following 2 h of incubation, although no significant difference was detected (Figure 3). Furthermore, no significant differences were recorded when comparing the experimental groups with their respective positive controls. 

Following 24 h, a significantly decreased acrosome integrity was observed in the *E. coli* positive control in comparison with the negative control (*p* < 0.01). Neither CUR nor KAN were able to significantly stabilize the acrosomal structures against the detrimental effects of bacteria present in the respective co-cultures. 

### 3.4. Mitochondrial Membrane Potential

**Figure 4:**
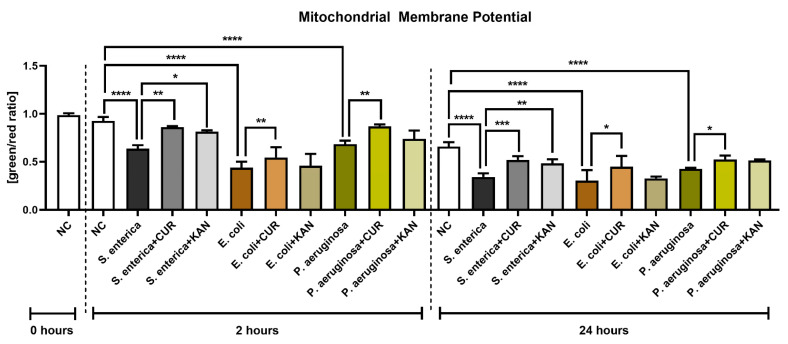
The effect of curcumin and kanamycin on rooster sperm mitochondrial membrane potential (green/red ratio]) during induced bacterial contamination. *p* < 0.05 (*), *p* < 0.01 (**), *p* < 0.001 (***), *p* < 0.0001 (****). NC—negative control, CUR—curcumin, KAN—kanamycin.

The presence of bacteria, particularly *E. coli*, led to a significant decrease in mitochondrial membrane potential as compared with the bacteria-free negative control after 2 h of co-culture (*p* < 0.0001; Figure 4). On the other hand, CUR supplementation in all relevant experimental groups led to a significant stabilization of the mitochondrial activity in comparison to their respective positive controls (*p* < 0.01). The presence of KAN led to a significant improvement only in the experimental group exposed to *S. enterica*.

Mitochondrial deterioration in the presence of selected uropathogens remained significant following 24 h of culture in comparison with the negative control (*p* < 0.0001). Accordingly, CUR continued to stabilize mitochondrial activity throughout the in vitro culture, as evidenced by a significantly higher mitochondrial membrane potential in all experimental groups when compared to their respective positive controls (*p* < 0.05 with respect to *E. coli* and *P. aeruginosa*; *p* < 0.001 in the case of *S. enterica*). While KAN supplementation to the co-culture led to a higher mitochondrial activity in the experimental groups, a significant improvement in mitochondrial membrane potential was observed only in the case of *S. enterica* (*p* < 0.01 against the positive control).

### 3.5. DNA Integrity

**Figure 5:**
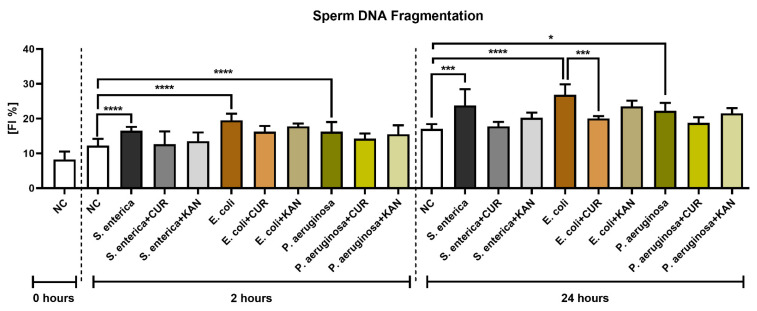
The effect of curcumin and kanamycin on rooster sperm DNA fragmentation (fragmentation index %) during induced bacterial contamination. *p* < 0.05 (*), *p* < 0.001 (***), *p* < 0.0001 (****). NC—negative control, CUR—curcumin, KAN—kanamycin.

The TUNEL assay revealed a significant decline in sperm DNA integrity in all control groups exposed to the uropathogenic bacteria (*p* < 0.0001; Figure 5) following 2 h of culture. No significant differences in sperm DNA stability were recorded in the experimental groups. 

The extent of sperm DNA damage remained significantly elevated in the positive controls following 24 h of culture (*p* < 0.05 for *P. aeruginosa*; *p* < 0.001 for *S. enterica*; *p* < 0.0001 for *E. coli*). Significant sperm DNA stabilization following CUR supplementation was observed in the experimental groups exposed to *S. enterica* (*p* < 0.01) and *E. coli* (*p* < 0.001). No significant changes in sperm DNA integrity were observed in the experimental groups administered with KAN.

### 3.6. Reactive Oxygen Species (ROS) Production

**Figure 6:**
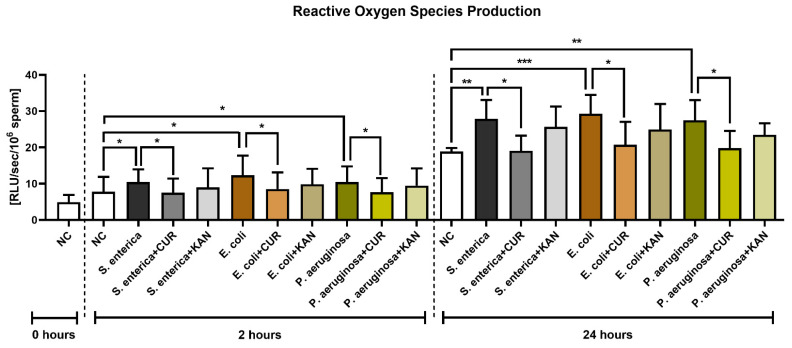
The effect of curcumin and kanamycin on ROS levels (RLU/s/10^6^ sperm) during induced bacterial contamination. *p* < 0.05 (*), *p* < 0.01 (**), *p* < 0.001 (***). NC—negative control, CUR—curcumin, KAN—kanamycin.

The presence of *S. enterica*, *E. coli* and *P. aeruginosa* in the positive controls was accompanied by increased ROS generation (*p* < 0.05) early on in the sperm–bacteria co-culture (2 h; Figure 6). While no significant differences were recorded following KAN supplementation, a significant decline in ROS amounts was observed in all experimental groups supplemented with CUR (*p* < 0.05).

ROS production remained significantly elevated in all positive controls in comparison to the negative control following 24 h of culture (*p* < 0.01 with respect to *S*. *enterica* and *P. aeruginosa*; *p* < 0.001 in the case of *E. coli*). CUR continued to exhibit its antioxidant effects, as evidenced by significantly lower ROS levels in the experimental groups when compared to their respective positive controls (*p* < 0.05). No significant differences were recorded amongst the experimental groups supplemented with KAN and their respective positive controls.

### 3.7. Bacteriological Analysis

**Figure 7:**
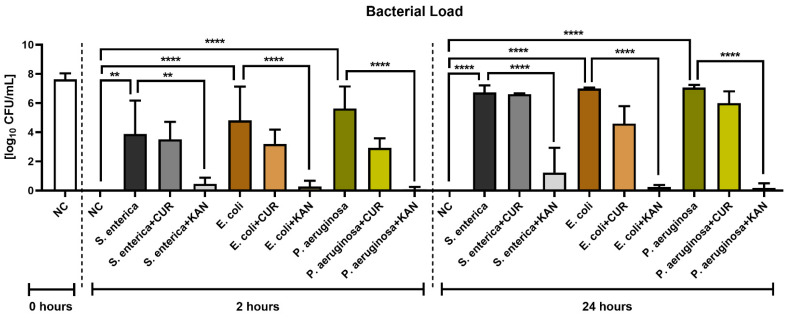
The effect of curcumin and kanamycin on bacterial load (log_10_ CFU/mL) during induced bacterial contamination. *p* < 0.01 (**), *p* < 0.0001 (****). NC—negative control, CUR—curcumin, KAN—kanamycin.

Figure 7 reveals the antimicrobial effects of CUR and KAN during in vitro induced bacterial contamination. A significant decrease in the growth of all selected uropathogens was observed in the experimental groups supplemented with KAN after 2 h (*p* < 0.01 with respect to *S. enterica*; *p* < 0.0001 in the case of *E. coli* and *P. aeruginosa*) and 24 h (*p* < 0.0001). While CUR supplementation led to a lower bacterial load in all experimental groups in comparison to their respective positive controls, no significant bacteriostatic effect of the biomolecule was observed. 

## 4. Discussion and Conclusions

Until recently, the impact of bacteria on sperm function has been underestimated, presumably due to only a short period of interaction between these cells during ejaculation. Nevertheless, if a contaminated sample is processed and diluted in buffered media designed to nourish and support male gametes, an environment will be created where bacteria and spermatozoa compete to sustain their vitality. In order to avoid the loss of semen samples suitable for AI and to decrease the risk of disease transmission to hens, readily available strategies to prevent or manage bacterial contamination during semen storage are necessary. In this study, we focused on three bacteria that have been repeatedly associated with infectious outbreaks in poultry production and which may contribute to decreased semen quality and fertility in roosters [35].

*Salmonella* spp. are frequently occurring uropathogens in poultry and poultry products. In addition, these bacteria have been found in semen from Vanaraja roosters [36] and turkeys [10], as well as in the cloacae, vaginas and uteri of hens [14,37], suggesting that their transmission may occur either through AI or via the passage of excreta from the cloaca [7]. In accordance with our results, Haines et al. [9] found that rooster spermatozoa co-incubation with *Salmonella* spp. at a concentration of 10^6^ cells/mL caused an immediate decline in sperm motion behavior. As observed by electron microscopy, the bacteria have high affinities to the sperm midpiece and tail, which could directly impact sperm motility [38]. What is more, it was reported that *Salmonella* spp. survive the freeze–thaw process to which turkey ejaculates are subjected, increasing the risks of semen cross-contamination during cryostorage [39].

In the meantime, *E. coli* is the most common bacterium found in poultry semen, as reported by several studies on roosters [36,40,41] and turkeys [5,6,18], with a negative impact on sperm motion and fertilization ability. In addition, it was noted by Mezhoud et al. [41] that the reproductive tract of broiler breeding roosters may also contain extended-spectrum β-lactamase (ESBL)-producing *E. coli* which may be transmitted to semen, which represents a concerning factor regarding the dissemination of ESBL-producing *E. coli* in poultry production. In agreement with our data, in vitro designed contamination of poultry semen by *E. coli* in earlier studies led to abrupt inhibition of sperm functional activity, particularly when the bacterial load exceeded 10^6^ CFU/mL [9,41].

Similar to *Salmonella* spp. and *Escherichia* spp., *Pseudomonas* spp. are common representatives of the seminal bacterial flora [6,36]. Moreover, as reported by Perek et al. [40], the bacteria were present in over a quarter of semen samples collected from cockerels in Israeli farms, with a persistent recurrence during follow-up semen collection and high potential to infest female reproductive organs following insemination. The presence of *P. aeruginosa* raises increasing concerns in avian pathology due to its emerging resistance to antibiotics and disinfectants [42]. Despite this fact, the effects of the bacterium on sperm functionality are the least studied amongst the uropathogens chosen for our experiments. Previous reports on mammalian species have unraveled that *P. aeruginosa* concentrations greater than 10^6^ CFU/mL are associated with a reduction in sperm kinetics, premature onset of capacitation and the subsequent inability of spermatozoa to accomplish fertilization [43].

A progressive loss of all sperm quality parameters was observed in all samples exposed to the bacteria, whilst this was negatively correlated with the increase in bacterial load. Detrimental effects of bacteria on the structures critical for sperm function and survival have been reported in different farm animals [11,12,13,31,43], including poultry [5,9,18,41]. In this regard, it shall be noted that all of the uropathogens selected for this study are G^-^ bacteria, known to contain adhesive pili [44] with a high affinity to mannose receptors located on the surface of sperm [45]. Bacterial adherence mediated by pili causes disintegration of the sperm membrane and sperm immobilization [46], as observed in our experiments. Accordingly, the process of sperm agglutination and immobilization may be accompanied by the disruption of the mitochondrial oxidative phosphorylation crucial for sperm movement [47], as observed with the rapid loss of mitochondrial membrane potential detected by the JC-1 assay. Another characteristic feature of G^-^ bacteria is their ability to secrete lipopolysaccharide (LPS), defined as a prototypical endotoxin, which triggers the expression of pro-apoptotic genes and decreases the intensity of the protein phosphorylation needed for sperm movement [48,49]. LPS is also a powerful pro-oxidant, exposure to which leads to ROS overproduction, with a concomitant decrease in sperm motility in humans [48] and boars [50], complementing our data. 

A deeper look at the precise effects of the selected bacteria on sperm function reveals that *E. coli* exhibited more detrimental effects on all studied sperm quality characteristics in comparison to *S. enterica* and *P. aeruginosa*. This may be explained by additional spermatotoxic behavior of *E. coli* mediated by the release of α-hemolysin, which forms pores in the sperm cell membrane, leading to a faster loss of membrane and acrosome integrity [51]. Furthermore, the highest amounts of ROS were observed in the positive control exposed to *E. coli*, which may have been side-effects of hemolysin action [52]. Finally, an almost non-existent sperm motility and mitochondrial activity accompanied by the highest degree of disruption to the membranous structures of rooster spermatozoa early on in the sperm–bacteria co-culture may have been caused by the sperm agglutinating factor and the sperm immobilizing factor secreted by *E. coli*. Both molecules have been reported to inhibit Mg^2+^-dependent ATPase [53] and to promote premature acrosome reactions by causing ionic imbalances in sperm cells [54]. 

Our results show that CUR proved to be a better motility-preserving agent than KAN in the case of *S. enterica* and *P. aeruginosa*, while KAN acted more effectively when spermatozoa were exposed to *E. coli*. The motility-preserving behavior of KAN has been previously reported by Sexton et al. [32], according to whom supplementation of a poultry semen extender with 31.2 µg/mL of KAN resulted in the better motility and fertility of stored rooster sperm in comparison to ampicillin or tobramycin. Nevertheless, these results must be treated with caution, since higher KAN concentrations (80 µg/mL) led to decline in in vitro motility in rabbit [31] and bull [55] spermatozoa. Furthermore, it has been suggested that high KAN concentrations could promote testicular oxidative stress by increasing ROS generation, followed by lipid peroxidation and alterations to the membrane fluidity of germ cells [56]. Inversely, previous reports observed that CUR could improve the motility and overall viability of extended or cryopreserved spermatozoa of several mammalian species [23,24,25,26,27], most likely through its ability to offer protection to the membranous structures or its remarkable antioxidant behavior. As suggested by earlier studies, the chemistry of CUR enables it to directly interact with the lipid bilayer of sperm membranes and thus prevent alterations to the plasmalemma and acrosome [23,26,57], while stimulating mitochondrial activity, leading to a higher preservation of sperm vitality during short- or long-term semen storage [23,58,59].

As discussed earlier, bacterial infestation of semen may lead to cell disintegration and death by ROS overproduction, ATP reduction and release of pro-inflammatory cytokines [60]. Interestingly, KAN was not fully able to protect spermatozoa against the loss of membrane fluidity and acrosomal disintegration. In this regard, it is of relevance to mention that high doses of aminoglycosides may cause multiple forms of cell death in other mammalian cells. Nevertheless, no distinct loss of cell viability was observed in the experimental groups exposed to bacteria and KAN; hence, we may suggest that any beneficial or detrimental effects of antibiotics may by and large depend on their concentration and time of exposure [61]. On the other hand, CUR can easily enter the intracellular space and exhibit anti-inflammatory effects mediated by the inhibition of tumor necrosis factor alpha (TNF-α) [62] and fortification of defense mechanisms against stress [57].

In this study, significant ROS overproduction was recorded in all positive controls. According to Fraczek and Kurpisz [63], bacteria and their toxins may directly or indirectly induce oxidative damage to male reproductive cells through the stimulation of oxidases, respiratory burst caused by activated leukocytes or excessive lipid peroxidation. Interestingly, KAN supplementation did not cause a significant decrease in ROS levels in the co-culture. What is more, it has been reported that under certain circumstances aminoglycosides may induce oxidative stress in healthy cells as a side-effect of their bactericidal action [64]. Hence, appropriate antioxidant supplementation should be considered to prevent such undesirable effects of antibiotics during semen storage. In the meantime, we detected a notable decline in ROS in the experimental groups exposed to CUR. This is in line with previous evidence on the ability of CUR to prevent the production of superoxide and hydroxyl radicals through intervention with the Fenton reaction [65,66]. Another reason for the improved oxidative profiles of the experimental groups exposed to CUR may lie in a stabilization of intrinsic ROS-scavenging molecules, as suggested by earlier studies on male gametes under in vitro induced stress conditions [57,66,67].

A frequently observed consequence of bacterial infestation is elevated damage to sperm DNA. Accordingly, significant DNA fragmentation was recorded in the positive controls, particularly in the case of *E. coli*. Parallel to our observations, high proportions of spermatozoa with damaged DNA were observed in ejaculates contaminated with *Escherichia* spp. or *Pseudomonas* spp. in turkeys [5], rams [13], boars [11] and humans [68]. It has been hypothesized that increased sperm DNA fragmentation in the presence of bacteria may be caused by oxidative insults to DNA molecules as an accompanying phenomenon of bacterial contamination [69]. This theory has been fortified by positive correlations between extent of sperm DNA damage and bacterial load and ROS levels in previous studies [5,12,13,31,47,60]. Additional loss of DNA stability could also be mediated by bacterial endotoxins known to trigger cell death accompanied by DNA disintegration [70]. In this study, sperm exposure to KAN led to higher sperm DNA fragmentation when compared to the groups supplemented with CUR. Recent studies indicate that aminoglycosides may trigger sperm apoptosis through oxidative mechanisms, accompanied by the loss of DNA integrity [71,72]. Inversely, our results gathered from the TUNEL assay indicate that CUR possesses DNA-protective properties, most likely thorough its direct ROS-quenching abilities, which prevent excessive amounts of oxidative by-products reaching the paternal genetic information and causing irreparable DNA breakage. Similar beneficial effects of CUR were also reported in bulls [23,66], rabbits [24,31], rats [58], dogs [65] and humans [57].

The bacteriological assessment of the control and experimental groups revealed that KAN performed better against the growth of all bacteria in comparison with CUR. Indeed, aminoglycosides, such as gentamicin and kanamycin, are currently the preferred antibiotic supplements to semen extenders and cryopreservation media in farm animals [11,31,32,73,74,75,76]. Nevertheless, due to alarmingly increased bacterial tolerance and/or resistance towards antibiotics, their use in animal reproduction science needs to be treated with caution. Gross et al. [77] concluded that *E. coli* found in 50% of marine birds and 25.5% of mammals was resistant to 14 antibiotics or antibiotic combinations, including gentamicin (10 µg) and kanamycin (30 µg). What is more, McMillan et al. [78] studied the ability of *S. enterica* and *E. coli* strains to mobilize three different kanamycin-resistance Col plasmids (KanR plasmids), which may support the spread of resistance to KAN in these uropathogens. Meanwhile, *P. aeruginosa* is well known to tolerate numerous antimicrobials and to develop multidrug resistance in clinical settings, which complicates anti-pseudomonal chemotherapy [79]. According to Faisal and Salman [80], aminoglycosides had variable efficiency against *E. coli* isolated from the semen of infertile subjects. Furthermore, Salman et al. [81] reported a higher resistance rate of *Salmonella enterica* serovar Typhi against aminoglycosides in patients seeking fertility treatment. In bulls, resistance to all tested antibiotics was identified in 22% of the microorganisms isolated from semen, including *Escherichia* spp. and *Pseudomonas* spp. [82]. Similar resistance patterns were also observed in semen samples collected from boars [83] and rams [13]. All in all, evidence gathered from this study and previous reports highlights the need for regular bacteriological assessment of ejaculates for AI, which may assist in the selection of appropriate antibiotics in order to avoid future complications associated with bacterial resistance.

While CUR was less effective in the inhibition of bacterial growth in this study, a decrease in bacterial load was observed in all experimental groups exposed to the biomolecule. Several earlier reports have shown that CUR presents broad-spectrum antibacterial activity and strong biological activity against a variety of bacteria, including potential uropathogens, such as *E. faecalis* [31], *Acinetobacter baumannii* [84], *P. aeruginosa* [29], *Klebsiella pneumoniae* [85], *S. aureus* [86] and *E. coli* [87]. The mechanisms underlying such behavior of CUR are multivariable and often employed simultaneously. CUR can inhibit bacterial growth by targeting bacterial cell walls or plasma membranes, DNA, proteins and other cellular structures critical for bacterial survival [88]. A specific target of CUR is the quorum sensing system, which plays pivotal roles in bacterial virulence, adhesion to host receptors and biofilm formation [89]. Indirectly, CUR may also hinder bacterial infestation through its antioxidant properties [31] and phototoxicity under blue-light excitation [90]. What is more, CUR may act in symbiosis with various antimicrobial drugs [91] and increase bacterial sensitivity to β-lactam antibiotics, such as methicillin and penicillin [92]. 

In our case, the low antibacterial activity of CUR may be explained by the supplement dose for the semen extender, which was well below 1 mmol/L. The selection of adequate concentrations of any supplement to multicellular systems must respect the tolerance levels of all cell types involved in the co-culture. Since CUR is known for an emblematic dichotomy [93], higher concentrations of the biomolecule with more prominent antibacterial effects may have been spermatotoxic. Furthermore, it has been observed by Adamczak et al. [30] that G^-^ bacteria are less sensitive to CUR when compared to their G^+^ counterparts, most likely due to the presence of an outer membrane, which is by and large responsible for the resistance of G^-^ bacteria to several antibiotics. What is more, amongst the bacteria selected for the experiments in this study, *Salmonella* spp. were least affected by the presence of CUR. As reported by Marathe et al. [94], CUR was able to stimulate the defense mechanisms of *S. enterica*, enhancing its pathogenicity. As such, more studies are needed to establish adequate conditions in which the use of CUR would be beneficial. 

Finally, we may speculate that since CUR was not able to completely prevent or reverse the negative effects of bacterial contamination on male gametes when compared to KAN, its beneficial effects might have been primarily caused by its ability to counteract ROS overproduction and/or endotoxins released by the bacteria, leading to a more favorable in vitro environment for sperm survival. In the meantime, higher sperm quality in the experimental groups subjected to KAN treatment may have been accomplished by direct antibacterial properties of KAN, leading to the elimination of the detrimental effects of bacterial presence in the co-culture. As such, we may hypothesize that CUR could act as a potentially valuable supplement for rooster semen extenders in combination with appropriately selected and dosed antibiotics. Nevertheless, any synergism or antagonism among CUR and conventional antibiotics during semen storage should be studied in more detail.

## Data Availability

The data presented in this study are available on request from the corresponding author.
